# Advanced Killing Potential of Thymol against a Time and Temperature Optimized Attached *Listeria monocytogenes* Population in Lettuce Broth

**DOI:** 10.3390/biom11030397

**Published:** 2021-03-08

**Authors:** Dimitra Kostoglou, Parthena Tsaklidou, Ioannis Iliadis, Nikoletta Garoufallidou, Georgia Skarmoutsou, Ioannis Koulouris, Efstathios Giaouris

**Affiliations:** Laboratory of Food Microbiology and Hygiene, Department of Food Science and Nutrition, School of the Environment, University of the Aegean, 81400 Myrina, Lemnos, Greece; dimitra_kostoglou@outlook.com.gr (D.K.); nenatsak13@gmail.com (P.T.); johniliadis1995@gmail.com (I.I.); nik1595g@gmail.com (N.G.); georgiask95@hotmail.com (G.S.); fns13054@fns.aegean.gr (I.K.)

**Keywords:** *Listeria monocytogenes*, attachment, stainless steel, lettuce, disinfection, thymol, benzalkonium chloride, strain variability, microbial resistance, response surface methodology

## Abstract

Fresh vegetables and salads are increasingly implicated in outbreaks of foodborne infections, such as those caused by *Listeria monocytogenes*, a dangerous pathogen that can attach to the surfaces of the equipment creating robust biofilms withstanding the killing action of disinfectants. In this study, the antimicrobial efficiency of a natural plant terpenoid (thymol) was evaluated against a sessile population of a multi-strain *L. monocytogenes* cocktail developed on stainless steel surfaces incubated in lettuce broth, under optimized time and temperature conditions (54 h at 30.6 °C) as those were determined following response surface modeling, and in comparison, to that of an industrial disinfectant (benzalkonium chloride). Prior to disinfection, the minimum bactericidal concentrations (MBCs) of each compound were determined against the planktonic cells of each strain. The results revealed the advanced killing potential of thymol, with a concentration of 625 ppm (= 4 × MBC) leading to almost undetectable viable bacteria (more than 4 logs reduction following a 15-min exposure). For the same degree of killing, benzalkonium chloride needed to be used at a concentration of at least 20 times more than its MBC (70 ppm). Discriminative repetitive sequence-based polymerase chain reaction (rep-PCR) also highlighted the strain variability in both biofilm formation and resistance. In sum, thymol was found to present an effective anti-listeria action under environmental conditions mimicking those encountered in the salad industry and deserves to be further explored to improve the safety of fresh produce.

## 1. Introduction

*Listeria monocytogenes* is a major foodborne, facultative intracellular, human pathogenic bacterium that causes listeriosis, a relatively rare but particularly serious infection that is characterized by both high morbidity and high mortality for the vulnerable population groups such as the elderly, pregnant, and immunocompromised individuals [1]. Based on the latest available data for 2018, 14 foodborne outbreaks and 2549 invasive (severe) human cases of listeriosis were reported in the EU, with a case fatality rate of 15.6% and 229 recorded deaths [2]. That year, vegetables and juices and other products thereof were the food vehicles causing the most strong-evidence outbreaks. This is not surprising considering that the microorganism is easily killed by cooking, and thus the most dangerous foods are those that are contaminated and consumed without heating, such as fresh vegetables and salads [3]. Alarmingly, 1.5% of the ready-to-eat (RTE) salads tested in Europe in 2018 were reported as positive for this pathogen [2]. The persistence of *L. monocytogenes* in food processing areas (sometimes even for years) is believed to be linked, among others, to its ability to strongly attach to the surfaces of the equipment (e.g., tanks, cutting tables, and conveyor belts) and buildings (e.g., walls, ceilings, drains, and floors), creating robust biofilms on them that can later withstand the typically applied sanitization processes [4,5]. As with other microorganisms, the biofilm-forming ability of *L. monocytogenes* may depend on the specific strain(s) employed, including the inherent genotype ability, surface properties, serotype, and origin, and may also be significantly influenced by the surroundings (e.g., temperature, nutrients, pH, osmolarity), in addition to the physicochemical properties of the substratum and the time the cells have available to attach to it and develop the sessile structure [6,7,8,9,10,11]. Indeed, several studies have been occupied with the influence of environmental conditions (especially those of interest to the food industries) on the biofilm-forming ability of *L. monocytogenes* and on its subsequent susceptibility to disinfectants, with the temperature being included among the most studied extrinsic parameters [12,13,14].

Nowadays, novel, cost-efficient, and sustainable methods need to be developed and successfully implemented to combat detrimental biofilms, such as those formed by or containing pathogenic microorganisms, in various areas, including the food industry [15]. This is due to the increased resistance of such microbial communities to many of the available biocides [5], combined with the potentially toxic effects of some of the latter and/or their byproducts for the health and the ecosystem [16,17]. In this direction, phytochemicals have been widely explored as anti-biofilm agents in the past years, mainly due to their great chemical diversity, the relative ease of acquisition, and their multi-target antimicrobial action [18]. One of the well-studied plant antimicrobial compounds is thymol (THY), which is the main component of the essential oils (EOs) of thyme, oregano, and some other widely distributed plants in the Mediterranean region [19]. However, although several studies have been occupied with the anti-biofilm action of THY against many microorganisms, including *L. monocytogenes* [20,21,22,23,24], little is still known on the superiority (if any) of that compound or other phytochemicals over some other classical surface disinfectants [25,26,27]. Considering all the above, the main aim of the current study was to compare the effectiveness of THY to that of benzalkonium chloride (BAC), a well-known quaternary ammonium compound (QAC) widely used as biocide in many sanitizing formulations applied in industrial, health care, home, and cosmetics settings [28], against sessile *L. monocytogenes* bacteria under attachment conditions trying to simulate as much as possible those encountered in the salad industry. Those latter conditions were initially here optimized following response surface methodology (RSM) [29] to predict that incubation time and temperature combination favoring the attachment of a four-strain *L. monocytogenes* cocktail to stainless steel (SS) coupons placed fully immersed in diluted sterile lettuce broth (dLB). The involvement of each strain in the formation of the mixed sessile community and its antimicrobial recalcitrance was also monitored by a repetitive sequence-based polymerase chain reaction (rep-PCR) approach [30]. Overall, THY was found to present an effective anti-listeria action, while the strain variability in both biofilm formation and resistance is also highlighted. 

## 2. Materials and Methods 

### 2.1. L. monocytogenes Strains and Preparation of Their Mixed Working Suspension

The four bacterial strains used in this study were the *L. monocytogenes* AAL20066 (ser. 1/2a), AAL20074 (ser. 4b), AAL20105 (ser. 1/2c), and AAL20107 (ser. 1/2b), all isolated from fresh mixed salads and kindly provided by Dr. Andritsos (Athens Analysis Laboratories S.A.; Metamorfosi, Attica, Greece). All strains were kept frozen (at −80 °C) in Tryptone Soy Broth (TSB; Lab M, Heywood, Lancashire, UK) containing 15% glycerol and were resuscitated through streaking on to the surface of Trypticase Soy Agar (TSA; Condalab, Torrejón de Ardoz, Madrid, Spain) and incubating at 37 °C for 24 h (precultures). Working cultures were prepared by inoculating a colony from each preculture into 10 mL of fresh TSB and further incubating at 37 °C for 18 h. Bacteria from those final working cultures were sedimented by centrifugation (4000× *g* for 10 min at room temperature), washed twice with quarter-strength Ringer’s solution (Lab M), and finally suspended in the same solution, so as to present an absorbance at 600 nm (A_600 nm_) equal to 0.1 (ca. 10^8^ CFU/mL). Those adjusted saline suspensions of each strain were finally mixed together and used for the subsequent attachment experiments.

### 2.2. Preparation of the Lettuce Broth

A total of 2 kg of fresh lettuce were bought from a topical greengrocer and immediately transported to the laboratory where the external leaves were removed, keeping only the greener parts of the inner leaves, which were washed well in tap water, weighted (ca. 500 g) and then transferred to a household juicer (Multipress Automatic; Braun AG, Kronberg im Taunus, Germany). Collected juice (ca. 300 mL) was placed in a glass beaker, covered with parafilm, and heated at 60 °C (in a water bath) for 30 min to inactivate endogenous enzymes. Following heating, the juice was kept on ice for 10 min, placed in plastic falcon tubes (50 mL), and centrifuged at 2000× *g* for 15 min at 4 °C. The supernatant was carefully removed, vacuum filtered through paper filter disks (87 g/m^2^; Munktell Filter AB, Falun, Sweden), and the resulting filtrate was then sterilized by passing through microbiological syringe filters (0.2 μm diameter; Whatman, Buckinghamshire, UK). Sterile juice broth was stored at −80 °C till needed for the attachment experiments, on the day of which was diluted 1:20 with sterile distilled water. That dilution was performed to imitate those nutritional conditions potentially found in the salad industry, also considering that the further dilution of the medium (till 1:50) did not much affect the planktonic growth of the tested strains (data not shown).

### 2.3. Experimental Design to Study the Combined Influence of Time and Temperature on Attachment

Α central composite rotational design (CCRD) including 10 experiments, each one twice executed (i.e., 20 experiments in total) (Table 1), was applied to determine the putative interactive effects of the two independent factors, that is the incubation time (*X_1_*, varying between 14.1 and 81.9 h) and temperature (*X_2_*, varying between 3 and 37 °C) on the concentration of the attached/biofilm cells (Log_10_ CFU/cm^2^) of the four-strain *L. monocytogenes* cocktail on the SS coupons, as previously described [29]. That design also allowed to determine those values of the two factors that upon concurrently applied would maximize the final population density (Log_10_ CFU/cm^2^) of the attached/biofilm bacteria. Each independent factor was coded at five levels, −1.414, −1, 0, 1, and 1.414, according to the following equation:

(1) $$x_{i}=\;\frac{X_{i}-X_{0}}{\Delta X_{i}},\;\;x_{i}=1,\;2$$

where *x_i_* and *X_i_* are the dimensionless and the actual value of the independent factor *i*, *X*_0_ the actual value of the independent factor *i* at its zero level (central point), and *ΔX_i_* the step change of *X_i_* correlating with a unit alteration of the dimensionless value. 

Obtained (measured) data were submitted to least square regression analysis to acquire the parameters of the derived polynomial mathematical equation. To confirm the ability of the latter to accurately predict the combined influence of the two independent factors on the accumulation of the bacteria on the SS surfaces, four additional experiments were also performed examining different combinations of the two independent factors than those used to generate the model. 

### 2.4. Disinfectants and Other Chemicals

Thymol (THY) was bought from Penta Chemicals (Radiová, Prague, Czech Republic) (powder min. 99.0%, molar mass: 150.22 g/mol; product code: 27450-30100), while benzalkonium chloride (BAC) was purchased from Acros Organics (Thermo Fisher Scientific, Geel, Belgium) (liquid, alkyl distribution from C8H17 to C16H33; product code: 215411000). The stock solution of THY (10% *w*/*v*) was prepared in absolute ethanol, while that of BAC (1% *v*/*v*) in sterile distilled water. Both stock solutions were maintained at 4 °C for up to two weeks. All other chemicals and reagents used for the experiments were purchased from Merck KGaA (Darmstadt, Germany) unless otherwise stated. 

### 2.5. Determination of the Minimum Inhibitory and Bactericidal Concentrations (MICs, MBCs) 

The minimum inhibitory concentration (MIC) and minimum bactericidal concentration (MBC) of each disinfection chemical (i.e., THY, BAC) against the planktonic cells of each *L. monocytogenes* strain were specified using the broth microdilution and agar spot methods, respectively, as previously described [26]. Briefly, to calculate the MICs, broth cultures (in TSB), inoculated with bacteria (ca. 10^5^ CFU/mL) and also containing 10 different increasing concentrations of each chemical, were statically incubated at 37 °C for 24 h. The tested concentrations for THY ranged from 10,000 to 19.5 ppm (two-fold dilutions), while those for BAC from 1 to 10 ppm (in 1 ppm increments). The MIC of each chemical was determined as its lowest concentration inhibiting the visible bacterial growth (i.e., no increase in broth’s turbidity), while the MBC was calculated as its lowest concentration reducing the initial inoculum by at least 3 logs (≥99.9%). Each experiment was thrice repeated starting from independent bacterial cultures.

### 2.6. Attachment of L. monocytogenes to SS under Various Time and Temperature Combinations and Quantification of the Sessile and Planktonic Populations

The four-strain *L. monocytogenes* cocktail was left to attach to or form biofilms on rectangular SS coupons (3 × 1 × 0.1 cm, type AISI 304), as previously described [31]. Briefly, individual, cleaned, and sterilized coupons were fully and vertically immersed in 1:20 dLB (5 mL in glass tubes), which was also inoculated with the mixed bacterial suspension, so as to have an initial concentration of ca. 10^3^ CFU/mL. This setup was then incubated under the 10 different combinations of time (varying from 14.1 h to 81.9 h) and temperature (varying from 3 °C to 37 °C) as these had been predetermined by the applied CCRD (Table 1). Following attachment/biofilm formation, the loosely attached cells were removed from the surfaces by placing each coupon in 5 mL of quarter-strength Ringer’s solution, under agitation for 5 min. This rinsing procedure was repeated once more and then each coupon was placed into a new sterile glass test tube containing 6 mL of quarter-strength Ringer’s solution and 10 sterile glass beads (diameter 3.0 mm), where it was thoroughly vortexed for 2 min (at 3000 rpm using ZX3 Advanced Vortex Mixer (VELP Scientifica Srl, Usmate, Italy) in order to detach from surfaces the strongly attached/biofilm bacteria. These were finally enumerated by counting colonies on (duplicate) spread inoculated (100 μL) TSA plates, following 10-fold serial dilutions in quarter-strength Ringer’s solution, plating, and incubation at 37 °C for 24 h. Upon no appearance of colonies on TSA plates (in some of the experiments combining short times with low temperatures), and to lower the detection limit, 1 mL was spread plated on four Petri dishes (i.e., 250 μL/plate) resulting thus in a detection limit of 1 CFU/mL corresponding to an attached population of 0.9 CFU/cm^2^ (equal to −0.05 Log_10_ CFU/cm^2^), given that the total surface area of each SS coupon was 6.8 cm^2^. For each experiment and at the end of incubation, the mixed planktonic population (Log_10_ CFU/mL) found in the dLB where the coupons had been placed was also enumerated through plate counting. 

### 2.7. Disinfection of the Mixed Sessile Community and Calculation of the Log Reductions

The four-strain *L. monocytogenes* cocktail was initially left to attach to/form biofilm on the SS coupons incubated in dLB under time and temperature conditions previously determined/verified to maximize the concentration (Log_10_ CFU/cm^2^) of the sessile bacteria (i.e., for 54 h at 30.6 °C). At the end of this incubation, the loosely attached cells were removed from the surfaces, as previously described (Section 2.6), and the coupons were then placed in the appropriate aquatic disinfectant solution (5 mL in glass tubes). Each disinfectant was left to act for 15 min at 20 °C and tested at three different concentrations, based on the previous determination of the MBCs (Section 2.5). Thus, THY was applied at two, three, and four times more than its MBC (i.e., 312.5 ppm, 468.8 ppm, and 625 ppm, where MBC = 156.3 ppm), while BAC at 4.7, 11.7, and 23.3 times more its MBC (i.e., 14 ppm, 35 ppm, and 70 ppm, where MBC = 3 ppm). Sterile distilled water was used as the negative disinfection control. This also contained 0.6% v/v ethanol when THY was used as the disinfectant, given that this low ethanol concentration was the one existing in the highest tested concentration for that terpenoid (i.e., 625 ppm). Following disinfection, each coupon was removed from the disinfectant solution and placed in 5 mL of quarter-strength Ringer’s solution, under agitation for 5 min, to remove disinfectant residues and was then immersed for 10 min in a 10 mL-plastic falcon tube containing 6 mL of Dey-Engley (D-E) Neutralizing broth (Lab M) and 10 sterile glass beads (3 mm in diameter). Strongly attached/biofilm cells were removed from surfaces and enumerated, as previously described (Section 2.6). Plate counts were converted to Log_10_ CFU/cm^2^ and for each disinfectant and tested concentration, the logarithmic reductions (Log_10_ CFU/cm^2^) of bacteria following disinfection were calculated by subtracting the log_10_ of the survivors from that counted following disinfection with water (negative control). 

### 2.8. Recovery of L. monocytogenes Colonies and DNA Extraction 

In total, 100 colonies were randomly selected and recovered from the highest dilutions of the TSA plates used to enumerate the strongly attached/biofilm viable bacteria found on the SS coupons both before and after their disinfection and from those plates used to enumerate the planktonic bacteria existing in the surrounding dLB at the end of incubation (i.e., for 54 h at 30.6 °C). More specifically, 20 colonies were recovered following the quantification of the planktonic population (treatment A), while other 20 colonies represented the sessile population being found on the SS coupons at the end of incubation and just before disinfection (treatment B). The other 60 colonies were recovered after disinfection and following the quantification of the remaining viable sessile bacteria. For this, one intermediate sub-lethal concentration per disinfectant was selected (out of the three concentrations tested). In particular, 20 colonies were isolated after each disinfection treatment, that is disinfection with water (negative control), 468.8 ppm THY, or 35 ppm BAC (treatments C, D, and E, respectively). All isolated colonies were suspended in TSB (1 mL) containing 15% (v/v) glycerol and stored at –80 °C till the extractions of their DNAs.

The genomic DNA of each isolate (x100) was extracted following the enzymatic method described by Doulgeraki et al. [32], with some minor adaptations. Analytically, a loopful of the frozen suspension of each isolate was streaked on to the surface of TSA and incubated at 37 °C for 24 h (precultures). Working cultures were prepared by inoculating a colony from each preculture into 5 mL of fresh TSB and further incubating at 37 °C for 18 h. Bacteria from those final working cultures were harvested by centrifugation (4000 x g for 10 min at room temperature) and resuspended in 0.5 mL of buffer lysis solution (1 Μ sorbitol, 0.1 Μ ethylenediaminetetraacetic acid, pH 7.5) containing 25 mg/mL lysozyme (Applichem GmbH, Darmstadt, Germany) and 5 U mutanolysin (from *Streptomyces globisporus* ATCC 21553; Merck), and incubated at 37 °C for 2 h. Following this incubation period, the mixture was centrifuged (5000 x g for 10 min at 4 °C) and the new pellet was suspended in 0.5 mL of buffer solution (50 mM Tris-HCl, 20 mM EDTA, pH 7.4), 50 μL of 10% sodium dodecyl sulfate (SDS) were also added and incubated at 65 °C for 30 min. Each sample was then mixed with 0.2 mL of 5 Μ potassium acetate, placed on ice for 30 min, and centrifuged at 20,000 x g for 10 min at 4 °C. The supernatant was precipitated with 1 mL of ice-cold isopropanol and centrifuged at 10,000 x g for 15 min at 4 °C. Each new pellet was washed by suspending it in 1 mL of ice-cold absolute ethanol and centrifuging at 20,000 x g for 15 min at 4 °C. Each pellet was then air-dried (at 40 °C for ca. 1.5 h) and resuspended in 120 μL of Tris-EDTA (ΤΕ) buffer (10 mM Tris-HCl, 1 mM EDTA, pH 8). The absorbances of each DNA solution were finally measured at 260 and 280 to determine the concentration of the extracted nucleic acids and their purity [33]. A 5 μL aliquot of each extracted DNA sample was also submitted to electrophoresis (1.5% w/v TBE agarose gel; 50 V for 1 h) to verify its integrity, while the rest of each sample was maintained at –20 °C to be used as a substrate in the subsequent rep-PCRs.

### 2.9. Discrimination of L. monocytogenes Strains through Rep-PCR 

Each recovered isolate (×100) was discriminated/typed to the strain level following a previously described rep-PCR approach [30], with some minor adaptations. Briefly, the Kapa Taq PCR Kit with dNTPs (KK1016, 500 U; Kapa Biosystems, Wilmington, MA, USA) was used for the PCRs. Each reaction mixture contained 2.5 μL of 10× kapa Taq Buffer A (1.5 mM final MgCl_2_ concentration at 1×); 0.5 μL of 10 mM dNTP Mix (0.2 mM final concentration); 2.5 μL of 10 μΜ GTG_5_ primer (GTG GTG GTG GTG GTG; 1 μM final concentration); 3 μL of DNA template (≈ 300 ng); 0.2 μL of Kapa Taq DNA polymerase (5 U/μL) and 16.3 μL of high-performance liquid chromatography (HPLC) grade water (Applichem) to a total volume of 25 μL. Following their preparation, the mixtures were placed in a PeqStar 96 HPL Gradient Thermocycler (Peqlab, VWR International GmbH, Darmstadt, Germany). The PCR program consisted of an initial denaturation step at 95 °C for 5 min, followed by 30 cycles of denaturation at 95 °C for 30 s, primer annealing at 40 °C for 1 min, and primer extension at 72 °C for 8 min, and this was concluded by a final extension step at 72 °C for 16 min. For each recovered isolate, the rep-PCR protocol was twice repeated on different days. 

Resulting amplicons were separated in a 1.5% (w/v) Tris-Borate-EDTA (ΤΒΕ) agarose gel, also containing 0.05 μg/mL of ethidium bromide (EtBr), in 0.5× TBE buffer, at 50 V for 2 h, using the Mupid-One electrophoresis system (NIPPON Genetics EUROPE GmbH, Dueren, Germany). FastGene^®^ 100 bp DNA Ladder (110 μg/1 mL; MWD100, NIPPON Genetics) was used as the molecular weight marker (5 μL/well), while DNA bands were detected by visualizing gels after electrophoresis under UV trans-illumination using the Quantum ST4 gel documentation imaging system (Vilber Lourmat, Marne-la-Vallée, France). To be sure for the correct discrimination/typing of the isolates (×100), the rep-PCR amplicons of DNAs extracted from pure cultures of each one of the four *L. monocytogenes* strains were always loaded on each gel (as controls, ×4), together with and next to those amplicons resulting from the rep-PCRs using as substrates the DNAs of the recovered isolates (samples).

### 2.10. Statistics and Graphics 

Each attachment experiment included two replicate SS coupons and was twice repeated starting from independent bacterial cultures. Disinfection experiments also included two coupons, but these were thrice repeated. Bacterial counts (CFU/mL and CFU/cm^2^ for planktonic and sessile populations, respectively) were converted to logarithms before calculating the means and standard deviations. Experimental design and statistical analysis were performed using JMP™ v10 (SAS Institute Inc., Cary, NC, USA). Analysis of variance (ANOVA) was applied to estimate the significance of the derived model at a *p*-value of 0.05, while the good fit of the derived equation was also assessed by its adjusted regression coefficient (R^2^_adj_). In addition, the predictive ability of the model was evaluated by determining the bias and accuracy factors (B_f_ and A_f_, respectively) of the four additional confirmation experiments, together with that one predicted maximizing sessile population, as previously reported [34]. The contour plot of the predicted data was constructed using SigmaPlot for Windows v11 (Systat Software Inc., Chicago, IL, USA). Pearson correlation analysis was finally applied to determine any possible correlation existing between planktonic and sessile populations for the various tested time and temperature combinations.

## 3. Results

### 3.1. Combined Influence of Time and Temperature on Sessile and Planktonic Cell Numbers

The measured and predicted values for the concentrations of the attached populations (Log_10_ CFU/cm^2^), of the four-strain *L. monocytogenes* cocktail to SS coupons, at the various selected time and temperature combinations, are presented in Table 1. Thus, under the current test conditions, the measured sessile populations were found to range from –0.05 ± 0.00 Log_10_ CFU/cm^2^ to 5.69 ± 0.07 Log_10_ CFU/cm^2^. Regarding the lowest sessile population, this was always observed at those experiments where the incubation temperature was ≤8 °C, and in parallel the attachment time ≤48 h (i.e., experiments 6, 7, 10, and 16). Indeed, under such short time and low-temperature conditions, the attached cells recovered from surfaces were always lower than the detection limit of the plate counting method (i.e., 0.9 CFU/cm^2^). On the other hand, the maximum observed sessile counts were recorded upon incubation at 32 °C for 24 h (experiment 8), with seemingly no significant differences upon increasing the incubation time up to 72 h at that specific temperature (experiments 14 and 20). 

The above presented measured data were submitted to multiple regression analysis to derive the polynomial mathematical equation, which is shown in Table 2. Equation fitting was estimated by determining its square regression coefficient (R^2^), its adjusted value (R^2^_adj_), and *p*-value, which were 0.97, 0.96, and < 0.0001, respectively. Those values clearly demonstrate an acceptable agreement between the measured and predicted data. The derived model could thus well predict the combined effects of the two factors (i.e., time and temperature) on the attached SS coupons population (Log_10_ CFU/cm^2^) of the four-strain *L. monocytogenes* cocktail. 

**Figure 1:**
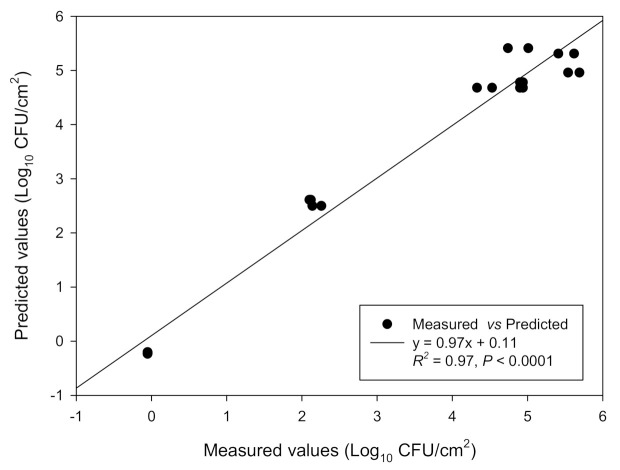
Linear relationship between the measured and predicted data for the concentrations of the attached to SS coupons populations (Log_10_ CFU/cm^2^) of the four-strain *L. monocytogenes* cocktail. The mathematical equation of the regression plot, together with its regression coefficient (*R^2^*), are also shown. Dots represent the mean values of all experiments included in the CCRD (*n* = 20; i.e., those shown in Table 1). For more clarity, the bars of standard deviations have been omitted.

Indeed, a satisfactory linear relationship was recorded between the measured and predicted data for the concentrations of the attached-to-SS coupons populations (Log_10_ CFU/cm^2^) of the four-strain *L. monocytogenes* cocktail (Figure 1). 

The determined values and fitting statistics (95% confidence limits, *p*-values, and t ratios) for the parameters of the derived model are presented in Table 3.

Following extraction of the mathematical equation, the application of the desirability function allowed the determination of that specific time and temperature combination which could maximize the sessile population of the four-strain *L. monocytogenes* cocktail to SS coupons. Thus, under those specified conditions (i.e., 54 h at 30.6 °C) the equation predicted a maximal value for the concentration of the attached/biofilm population equal to 5.65 ± 0.40 Log_10_ CFU/cm^2^. Indeed, the confirmation experiment performed under those specific conditions resulted in a measured attached/biofilm population of 5.46 ± 0.31 Log_10_ CFU/cm^2^ (Table 4).

**Figure 2:**
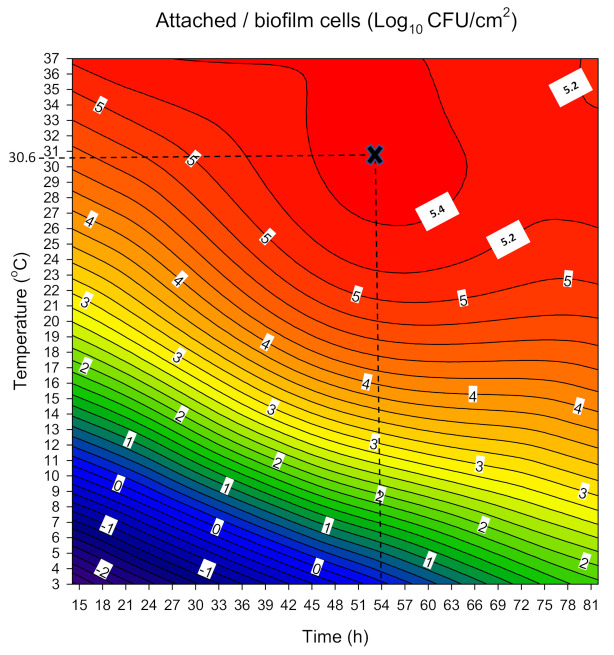
Contour plot describing the interactive influence of incubation time and temperature on the concentration of the attached SS coupons population (Log_10_ CFU/cm^2^) of the four-strain *L. monocytogenes* cocktail. This plot was constructed by considering the predictions of all experiments included in the central composite rotational design (CCRD) (*n* = 20; i.e., those shown in Table 1). Dotted lines illustrate time and temperature conditions predicted maximizing sessile population (i.e., 54 h at 30.6 °C).

The alterations in the concentration of sessile cells (Log_10_ CFU/cm^2^) as a function of the concurrent change of each possible pair of the two factors evaluated (i.e., time and temperature) are presented in the form of a contour plot in Figure 2. It is clear that the concentration of the attached/biofilm cells tends to be maximized (>5.4 Log_10_ CFU/cm^2^) at temperatures above 26 °C combined with incubation time ranging from approximately 45 h to 65 h. At the same time, the further increase of incubation temperature (>26 °C) seems to decrease the time needed for the cells to maximize their accumulation onto the surfaces. For instance, at 37 °C, attached cells surpass the density of 5.4 Log_10_ CFU/cm^2^ as early as 30 h of incubation. However, at this high temperature, the sessile population seems to slightly decrease earlier as incubation time passes (i.e., <5.4 Log_10_ CFU/cm^2^ from 60 h and more). This is probably due to detachment phenomena.

**Figure 3:**
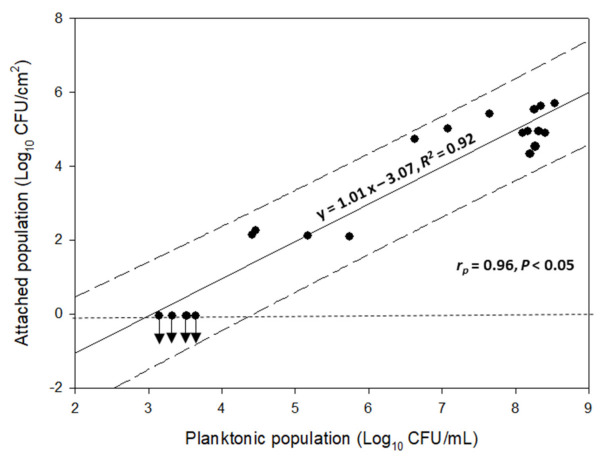
Correlation between the concentrations of the measured planktonic (Log_10_ CFU/mL) and attached populations to the SS coupons (Log_10_ CFU/cm^2^) of the four-strain *L. monocytogenes* cocktail. The solid line represents the linear regression equation, while the dashed parallel lines represent the prediction intervals (α = 0.95). The mathematical equation of the linear regression, together with its regression coefficient (*R*^2^), Pearson correlation coefficient (*r_p_*), and *p*-value are also presented. Dots represent the mean values of all experiments included in the CCRD (*n* = 20; i.e., those shown in Table 1). For more clarity, the bars of standard deviations have been omitted. The horizontal dotted line illustrates the detection limit of the plate counting method of the sessile cells (i.e., −0.05 Log_10_ CFU/cm^2^).

Figure 3 shows the positive linear correlation between the concentrations of the measured planktonic (Log_10_ CFU/mL) and attached populations to the SS coupons (Log_10_ CFU/cm^2^) of the four-strain *L. monocytogenes* cocktail. It is obvious that these two factors are related to each other with the increase of one factor leading to (or perhaps caused by) the increase of the other factor.

### 3.2. Calculation of MICs and MBCs of THY and BAC against Planktonic Cells and Disinfection of the Mixed Sessile Community

The MIC of THY was found equal to 78.1 ppm against all four *L. monocytogenes* strains, while a double concentration was always needed to kill their planktonic cells (i.e., MBC = 156.3 ppm). The MIC of BAC was found equal to 2 ppm against all *L. monocytogenes* strains, quite close to the MBC for that compound, which was determined at 3 ppm. 

**Figure 4:**
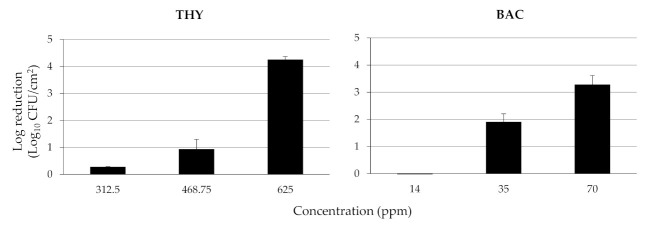
Log reductions of attached/biofilm cells on SS coupons (Log_10_ CFU/cm^2^), of the four-strain *L. monocytogenes* cocktail, following the 15 min disinfection exposure to either thymol (THY) or benzalkonium chloride (BAC), each applied at three different concentrations (ppm). The bars represent the mean values ± standard deviations (*n* = 6).

The log reductions of attached/biofilm population on SS coupons of the four-strain *L. monocytogenes* cocktail (5.46 ± 0.31 Log_10_ CFU/cm^2^), following the 15 min disinfection exposure to each chemical (i.e., THY, BAC) being applied at three different concentrations (ppm), are presented in Figure 4. As expected, log reductions increased as the chemicals’ concentrations increased, meaning that more bacteria died upon increasing the chemical’s concentration. More importantly, the results revealed the significant disinfection efficiency of THY, with a concentration of 625 ppm (= 4 × MBC), leading to almost undetectable (<0.95 Log_10_ CFU/cm^2^) viable bacteria (i.e., more than 4 logs reduction; 99.99% killing rate). On the other hand, the reduction did not exceed 3.5 logs even when BAC was applied at 23.3 times more than its MBC (i.e., 70 ppm).

### 3.3. Strain Variability on Planktonic Growth, Attachment, and Disinfection Resistance

**Figure 5:**
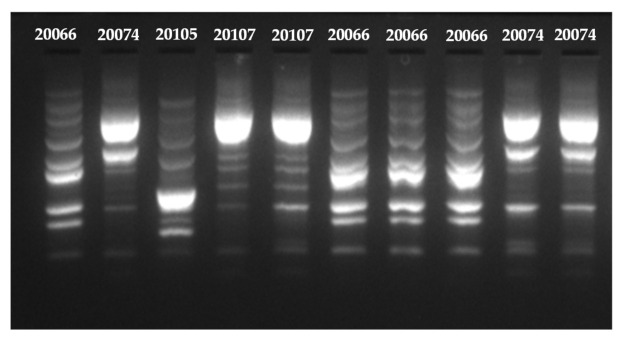
Characteristic repetitive sequence-based polymerase chain reaction (rep-PCR) profiles of 10 *L. monocytogenes* isolates, where it can be observed that each strain (AAL20066, AAL20074, AAL20105, and AAL20107) presents a distinct amplicon pattern enabling this way its easy discrimination from the other ones.

As expected, the four *L. monocytogenes* strains used in this study to prepare the cellular attachment cocktail (i.e., AAL20066, AAL20074, AAL20105, and AAL20107), were found to present distinct rep-PCR profiles (Figure 5), thus enabling their easy discrimination through the applied rep-PCR approach. After all, those strains had been deliberately chosen so that each belongs to a different serovar (i.e., 1/2a, 4b, 1/2c, and 1/2b, respectively).

**Figure 6:**
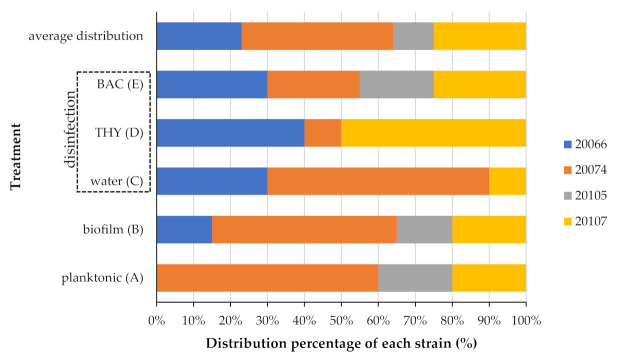
Distribution percentages (%) of each *L. monocytogenes* strain (i.e., AAL20066, AAL20074, AAL20105, and AAL20107) for each of the five examined treatments (i.e., A, B, C, D, and E; previously described in Section 2.8). The average overall distribution of each strain is also shown.

The contribution of each strain in the composition of the mixed planktonic population, which existed at the end of incubation (i.e., for 54 h at 30.6 °C) in the dLB in which the SS coupons had been placed as substrata for the bacterial attachment, in addition to that encountered in the mixed sessile communities found on the surfaces, both before and after their disinfection (i.e., 15-min exposure to water as control, 468.8 ppm THY, or 35 ppm BAC), are depicted in Figure 6. In general, the different strains were found to behave differently regarding their either planktonic or sessile growth and their disinfection resistance; the latter also found to be affected by the applied disinfectant. Thus, for instance, strain AAL20066 was not at all detected in the planktonic population (treatment A), whereas this strain still represented the 15% of the isolates (colonies) recovered from those plates used to quantify the attached/biofilm population (treatment B). As another example of this variability, this time on resistance, strain AAL20105 was not at all detected following disinfection with either water (control) or THY (468.8 ppm) (treatments C and D, respectively), whereas the 20% of colonies appearing on the plates following disinfection with BAC (70 ppm) belonged to that strain (treatment E). That heterogeneity in the behavior of each strain is also evident when someone considers the average overall distribution of each one in which, for instance, strain AAL20074 presented an appearance rate of 41%, whereas strain AAL20105 appeared approximately four times less exhibiting an average overall distribution equal to 11%.

## 4. Discussion

Various phytochemicals, either in pure form or as components of plant extracts, have been last years tested as anti-biofilm agents to overcome antimicrobial resistance (AMR) against various harmful microorganisms, including significant foodborne pathogenic bacteria, such as *Salmonella enterica*, *Campylobacter* spp., *L. monocytogenes* and *Escherichia coli* O157:H7 [35,36]. In this study, a natural terpenoid found in rich quantities in the EOs of thyme and other aromatic plants, already authorized as a food additive in many countries, THY, was tested against a sessile cocktail of four foodborne *L. monocytogenes* strains, all previously isolated from mixed salads and each belonging to a different serovar. Three of the four serovars here tested (i.e., 1/2a, 1/2b, and 4b) are known to cause the vast majority of human listeriosis cases [37]. Similarly, most of the *L. monocytogenes* strains isolated from foods and food processing environments belong to one of those three serovars, although the relative abundance of each serovar differs from that observed in clinical cases [37]. Strains belonging to serovar 1/2c are also pathogenic and isolated from retail foods, including RTE ones [38,39].

A mixed bacterial suspension, containing equal cell numbers for each strain, was initially left to attach to SS coupons incubated in sterile dLB under various time and temperature combinations, to extract a mathematic model which could be able to predict the density of the attached population (Log_10_ CFU/cm^2^) as a combined function of time and temperature, and as thus be able to define that specific combination of those two environmental factors (i.e., 54 h at 30.6 °C) that would maximize that density [40]. This last was desired so that the subsequent disinfection experiments could be executed following a worst-case scenario in which the environmental conditions would be quite favorable for the bacterial attachment. Surely, in addition to temperature, several other environmental factors could influence that attachment (e.g., pH, nutrients, osmolarity). Although some of those could be incorporated into the model, the reason for not doing so was because our primary aim was to comparatively evaluate the effectiveness of the two studied disinfectants (i.e., THY and BAC) against a well-established sessile *L. monocytogenes* population left to be formed in a specific plant-based growth medium, that is the dLB, and not to study the influence of environmental factors in general on attachment/biofilm formation by that pathogen. In addition, this incorporation could probably result in an inability for that specific CCRD design to accurately predict the combined (complex quite probably) influence of all those many interacting parameters. To deliver the model, RSM was applied, which is, in general, a collection of mathematical and statistical techniques based on the fit of a polynomial equation to a set of experimental data, with the ultimate objective of making statistical previsions [41]. The generation of large amounts of information from even a small number of experiments, decreasing thus time, labor, and expenses, is the main advantage of this multivariate technique, together with the possibility of evaluating the interaction effect between the tested variables on the studied response. This latter effect is not fully depicted when someone follows a one-variable-at-a-time approach, as is usually the case in most of the studies published so far regarding the influence of environmental factors on attachment and/or biofilm formation [42]. So far, RSM has mainly been used as a tool for optimization in analytical chemistry; however, it can be well applied whenever a response (e.g., biofilm formation), or even a set of responses of interest, may be influenced by several variables (e.g., environmental factors). Thus, this technique has also been used in the field of microbiology, specifically in inactivation applications [43,44], and, in recent years, also in biofilm research [31,45,46,47].

It is well-known that the nutrients that are available in a medium can affect both the attachment of the bacteria to surfaces and their subsequent sessile proliferation [7,8,14]. A diluted lettuce broth (dLB) was here used to simulate nutritional conditions potentially encountered within the fresh salad industry. However, it should still be noted that this broth was initially heated (at 60 °C for 30 min). Although this was just carried out to inactivate the endogenous enzymes of the plant tissue, this mild heating may slightly change the nutritional and physicochemical characteristics of that broth. However, if not inactivated, the action of those enzymes could still alter those parameters as well over time, considering that the lettuce broth was de facto impossible to be directly used on the day of its preparation. After all, previous studies that had also used vegetables’ broths as substrates for bacterial growth, have conducted similar (or even quite more intense) heating steps during their preparation [48,49,50]. Until now, however, few other studies have been published using such model food systems and dealing with the sessile behavior of *L. monocytogenes* [51,52], although these studies could better imitate the “real” scenario. Before diluting the lettuce broth (i.e., 1:20), this was also here sterilized through filtration. This sterilization was performed to simplify the experimental approach, choosing to initially work with pure (mono-species) cultures of the tested pathogen and be easier able to make observations and deliver conclusions. However, it should be noted that in the “real” world, bacteria belonging to different species and genera may be found together in the same niche, even together with other microorganisms (e.g., fungi, protozoa, bacteriophages), interacting with each other, with these intra/inter-species and inter-kingdom interactions be particularly evident in most of the natural biofilm communities, affecting their overall physiology and resistance [53,54]. 

It was here found that the increase of the incubation time above 45 h and in parallel of temperature above 30 °C had a favorable effect on the accumulation of the bacteria on the SS surfaces, with the sessile population being maximized following growth for 54 h at 30.6 °C. Surely, the temperature is one of the most significant factors affecting microbial growth, either planktonic or sessile. Not surprisingly, this is an environmental parameter widely investigated in biofilm research, including *L. monocytogenes*; the results obtained, however, are not always consistent. Thus, there are studies showing the increase of biofilm formation by that pathogen upon increasing the temperature toward that optimum for planktonic growth (i.e., 30–37 °C) [9,55,56], something that was also observed in this study, whereas other studies demonstrated an increase in sessile growth upon decreasing temperatures to sub-optimal ranges for planktonic growth, such as 15–20 °C [13] or even lower [57]. These differences are probably explained by the different strains employed in the various studies, combined with the rather complex nature of biofilm formation, even for mono-species consortia. Thus, the biofilm-forming ability of a given strain may be influenced by several other parameters, such as the morphological and physicochemical characteristics of the attachment substrate, available nutrients, shear stress, pH, and osmolarity, with interactive effects more often being observed between some of those parameters [58]. It is hence not strange for a given microbial strain to alter its biofilm-forming capacity in response to changing environmental conditions [59]. With all this in mind, a cocktail of four different strains was here employed in the attachment and the subsequent disinfection experiments.

The MICs that were here determined for the two tested chemicals (i.e., THY, BAC) were close to the ones having been previously reported against *L. monocytogenes*. Thus, the MIC of THY for that bacterial species is usually ranging between 78.1 ppm and 1024 ppm [22,23,60], while that of BAC is generally lower than 10 ppm, except for some BAC-tolerant isolates for which this value may be even higher than 20 ppm [61,62,63]. Although no critical breakpoints for disinfectant resistance have been defined, unlike for antibiotics, the four isolates here tested do not seem to present resistance to either of those two chemicals, based on the data available screening collections of more strains. The MBCs previously recorded for both chemicals are usually slightly higher compared to the respective MICs, considering that both present a strong bactericidal activity, something that was also here verified. Thus, the MBCs here recorded were equal to 156.3 ppm and 3 ppm, for THY and BAC, respectively. Following the calculation of the MBCs against the planktonic cells of each strain, each chemical was tested at three different concentrations, all higher than the MBCs, against the mixed sessile community on the SS surfaces. The results revealed the significant anti-biofilm potential of THY, considering that its application at just four times its MBC (i.e., 625 ppm) was sufficient to kill almost all sessile bacteria (> 4 logs reduction). On the other hand, the application of BAC at even 23.3 times more than its MBC (i.e., 70 ppm) reduced the sessile population by 3.28 logs. However, it should be noted that although sessile bacteria were here found to be quite more resistant than the planktonic ones (against both chemicals and especially against BAC), it is still alleviative that the application of BAC at the concentration this is usually applied in food industries (i.e., 200 ppm) resulted in the complete killing of the sessile population (data not shown). This is still not always the case for many other pathogenic bacteria being enclosed in biofilm structures. For instance, in a previous related study, the application of BAC at 200 ppm caused only a 1.5 log reduction of an *Staphylococcus aureus* biofilm population (> 10^7^ CFU/cm^2^) on polystyrene surfaces [26]. Another aspect, however, that should be always also considered is the potential for bacterial cells surviving disinfection to enter the viable but not-culturable (VBNC) state, being thus unable to be enumerated by traditional plating methods, such as those here applied. As it seems, this is not a so rare phenomenon and has also been described for *L. monocytogenes* following the action of QAC disinfectants [64,65] and some EOs [66]. If this is indeed the case, the log reductions here recorded might be even lower.

THY and other components of EOs are known to kill the microbial cells mainly due to their interaction with cellular membranes causing their collapse [19]. Considering the significantly better anti-biofilm potential of THY over the classical surface disinfectant that was here observed, this may be due to the better ability of the terpenoid to diffuse through the biofilm matrix, and thus, kill the cells. Noteworthily, in a previous related study, the hydrosol of the Mediterranean spice *Thymbra capitata*, consisting a plant mixture that also contains THY, was visualized in real-time, through confocal microscopy, to easily penetrate the biofilm structure of *S*. Typhimurium, killing much more quickly the enclosed bacteria, compared to BAC [25]. In another recent study, evaluating and comparing the disinfection efficiency of THY and BAC against preformed biofilms of either *S. aureus* or *Staphylococcus epidermidis*, it was again shown that the phytochemical presented a significant lower resistance coefficient (Rc) than the synthetic biocide, meaning that the required increase in its concentration to be equally effective against biofilm cells as this was against the planktonic ones was much lower compared to the synthetic biocide [26].

The involvement of each strain in the formation of the mixed sessile community and its antimicrobial recalcitrance was also here monitored by recovering many colonies (both before and after disinfection) and discriminating/typing them to the strain level through rep-PCR. This is a rather classical and broad range genomic fingerprinting technique that has been widely applied to genotype bacteria through PCR amplification of various lengths fragments of their genomic DNA, using primers that are complementary to repetitive sequences occurring in prokaryotic genomes and resulting in strain-specific amplicon patterns [67]. Results revealed heterogeneity in the behavior of each strain, with the recorded distribution percentages found to vary depending on the mode of growth (planktonic *vs* biofilm) and the tested disinfectant. This strain variability in biofilm formation and disinfection resistance is not something peculiar, and it has to do with the inherent differences in molecular and physiological aspects of microbial behavior between the different strains [68]. In addition, this has already been previously observed in multi-strain biofilms of *L. monocytogenes* and other species submitted or not to disinfection [69,70,71]. Considering that the planktonic cells of all here tested strains presented equal sensitivity to the two antimicrobials (based on the MIC and MBC results) and in parallel displayed similar planktonic growth rates in the dLB (data not shown), the differences observed in the strains’ prevalence at the different examined treatments should be associated with other parameters affecting sessile growth and resistance (e.g., production of extracellular polymeric substances [EPS], motility, coaggregation), such as those determining the final placement of each strain within the developed sessile structure, and thus its antimicrobial exposure, if this is not a coincidence. It should be finally stated that although someone could have tested the biofilm-forming ability and resistance of each strain one by one, the rep-PCR approach that was here followed allowed the evaluation of any strain variability with respect to those attributes somehow in situ. This is because any observation and conclusion made on single-strain cultures could not be safely extended to a mixed-strain cocktail, since in that latter case, each strain can interact with each other possibly affecting its sessile and resistance behavior. Indeed, a previous relevant study has shown quite complex patterns of bacterial interactions within mixed-culture *L. monocytogenes* biofilms [70].

## 5. Conclusions

A well-known natural terpenoid of plant origin (i.e., THY) was found to present strong killing efficiency against a sessile population of a multi-strain *L. monocytogenes* cocktail found on SS surfaces. Thus, its application at 625 ppm (= 4 × MBC) resulted in the almost elimination of the attached bacteria (> 4 logs reduction). On the other hand, the application of a widely used synthetic biocide (i.e., BAC) at a concentration that was more than 20 times higher than its MBC (i.e., 70 ppm) caused a significantly lower reduction (3.28 logs). The attached population was quite more resistant to the action of both disinfectants compared to the planktonic cells. That population has been initially optimized for the maximum cellular density (5.46 ± 0.31 log_10_ CFU/cm^2^), leaving bacteria to attach to surfaces under the most favorable time and temperature incubation conditions (i.e., for 54 h at 30.6 °C). Those conditions were determined by applying response surface modeling, delivering a mathematical model capable of predicting the interactive influence of both factors (i.e., time and temperature) on the accumulation of the pathogenic bacteria on the SS surfaces, under other environmental conditions mimicking those encountered in the salad industry. Strain variability in biofilm formation and resistance was also recorded. This study, hopefully together with some future studies that will also consider and incorporate the potentially rich natural microbiota found in these food environments, offer knowledge on the sessile behavior of this important foodborne pathogenic bacterium, highlighting an alternative sustainable way for its elimination. Phytochemicals such as THY deserve to be further studied to improve the safety of the fresh produce limiting possibilities of infections following their consumption. 

## Figures and Tables

**Table 1:** Experimental design with real and coded values of the two independent factors (i.e., time and temperature) evaluated for their influence on the attached to stainless steel (SS) coupons population (Log_10_ CFU/cm^2^) of the four-strain *L. monocytogenes* cocktail. Measured and predicted values of Log_10_ CFU/cm^2^, as defined for each individual experiment, are also shown.

Experiment	Independent Factors ^a^		Response ^b^	
	Incubation Time (h) (*Χ*_1_)	Incubation Temperature (°C) (*Χ*_2_)	Attached Population (Log_10_ CFU/cm^2^)	
			Measured	Predicted
1	14.1 (−1.414)	20 (0)	2.12 ± 0.22	2.61 ± 0.25
2	72 (1)	8 (−1)	2.26 ± 0.32	2.50 ± 0.25
3	48 (0)	37 (1.414)	5.01 ± 0.20	5.41 ± 0.25
4	48 (0)	37 (1.414)	4.74 ± 0.50	5.41 ± 0.25
5	72 (1)	8 (−1)	2.14 ± 0.34	2.50 ± 0.25
6	24 (−1)	8 (−1)	−0.05^c^ ± 0.00	−0.20 ± 0.25
7	48 (0)	3 (−1.414)	−0.05^c^ ± 0.00	−0.23 ± 0.25
8	24 (−1)	32 (1)	5.69 ± 0.07	4.96 ± 0.25
9	24 (−1)	32 (1)	5.54 ± 0.16	4.96 ± 0.25
10	48 (0)	3 (−1.414)	−0.05^c^ ± 0.00	−0.23 ± 0.25
11	48 (0)	20 (0)	4.94 ± 0.09	4.68 ± 0.22
12	81.9 (1.414)	20 (0)	4.90 ± 0.12	4.78 ± 0.25
13	14.1 (−1.414)	20 (0)	2.10 ± 0.30	2.61 ± 0.25
14	72 (1)	32 (1)	5.62 ± 0.02	5.31 ± 0.25
15	48 (0)	20 (0)	4.90 ± 0.38	4.68 ± 0.22
16	24 (−1)	8 (−1)	−0.05^c^ ± 0.00	−0.20 ± 0.25
17	48 (0)	20 (0)	4.33 ± 1.04	4.68 ± 0.22
18	81.9 (1.414)	20 (0)	4.94 ± 0.20	4.78 ± 0.25
19	48 (0)	20 (0)	4.53 ± 0.75	4.68 ± 0.22
20	72 (1)	32 (1)	5.41 ± 0.04	5.31 ± 0.25

^a^ Codes values are shown in parentheses. ^b^ Measured values are means ± standard deviations, while predicted ones are means ± standard errors. ^c^ Detection limit of the plate counting method.

**Table 2:** Polynomial mathematical equation and statistical parameters (*R*^2^, *R*^2^*adj,* and *p-value*) characterizing the influence of the two independent factors evaluated (i.e., time and temperature) on the attached to SS coupons population (Log_10_ CFU/cm^2^) of the four-strain *L. monocytogenes* cocktail.

Response	Polynomial Equation	*R* ^2^	*R* ^2^ *_adj_*	*p*
Log_10_ CFU/cm^2^	4.68 + 0.77 × t + 1.99 × T − 0.59(t × T) − 0.49 × t^2^ − 1.04 × T^2^	0.97	0.96	<0.0001

Refers to the coded values of the independent factors.

**Table 3:** Calculated values and fitting statistics for the parameters of the polynomial mathematical equation predicting the attached to SS coupons population (Log_10_ CFU/cm^2^) of the four-strain *L. monocytogenes* cocktail as a function of time (t) and temperature (T).

Parameter	Estimated Value a	95% Confidence Limits		*p*-Value	t Ratio
		Lower	Upper		
Intercept	4.68 ± 0.22	4.20	5.15	<0.0001	21.32
t	0.77 ± 0.11	0.53	1.00	<0.0001	6.98
T	1.99 ± 0.11	1.76	2.23	<0.0001	18.18
t * T	−0.59 ± 0.16	−0.92	−0.25	0.002	−3.79
t^2^	−0.49 ± 0.15	−0.80	−0.18	0.004	−3.38
T^2^	−1.04 ± 0.15	−1.35	−0.73	<0.0001	−7.18

^a^ Values are means ± standard errors.

**Table 4:** Confirmation experiments of the mathematical equation describing the concentration of the attached to SS coupons population (Log_10_ CFU/cm^2^) of the four-strain *L. monocytogenes* cocktail as a function of time and temperature. Measured and predicted values of sessile populations (Log_10_ CFU/cm^2^) for each individual experiment, together with the bias and accuracy factors of the model, are also shown. The 5th experiment was the one executed under conditions (i.e., 54 h at 30.6 °C) predicted to result in the maximum sessile population (5.65 ± 0.40 Log_10_ CFU/cm^2^).

Experiment	Independent Factors ^a^		Response ^b^	
	Incubation Time (h) (*Χ_1_*)	Incubation Temperature (°C) (*Χ_2_*)	Attached Population (Log_10_ CFU/cm^2^)	
			Measured	Predicted
1	36 (−0.5)	26 (0.5)	5.39 ± 0.14	5.05 ± 0.40
2	60 (0.5)	26 (0.5)	5.40 ± 0.09	5.52 ± 0.40
3	66 (0.75)	11 (−0.75)	2.96 ± 0.37	3.22 ± 0.40
4	66 (0.75)	29 (0.75)	5.58 ± 0.05	5.55 ± 0.40
5 (max)	54 (0.25)	30.6 (0.89)	5.46 ± 0.31	5.65 ± 0.40
			Factors	
			Bias	1.01
			Accuracy	1.04

^a^ Codes values are shown in parentheses. ^b^ Values are means ± standard deviations.

## Data Availability

The data presented in this study are available on request from the corresponding author.
